# Diencephalic syndrome in childhood, a challenging cause of failure to thrive: miniseries and literature review

**DOI:** 10.1186/s13052-022-01316-4

**Published:** 2022-08-17

**Authors:** Sandra Trapani, Barbara Bortone, Martina Bianconi, Chiara Rubino, Iacopo Sardi, Paolo Lionetti, Giuseppe Indolfi

**Affiliations:** 1 Department of Health Sciences, Pediatric Unit, Meyer Children’s University Hospital, Viale Pieraccini 24, 50139 Florence, Italy; 2 Meyer Children’s Hospital, Viale Pieraccini 24, 50139 Florence, Italy; 3 Pediatric Unit, Meyer Children’s Hospital, Viale Pieraccini 24, 50139 Florence, Italy; 4 Neuro-Oncology Unit, Department of Pediatric Medicine, Meyer Children’s Hospital, Florence, Italy; 5 NEUROFARBA Department, Gastroenterology and Nutrition Unit, University of Florence, Meyer Children’s Hospital, Viale Pieraccini, 24, 50139 Florence, Italy; 6 NEUROFARBA Department, Pediatric Unit, Meyer Children’s Hospital, University of Florence, Viale Pieraccini 24, 50139 Florence, Italy

**Keywords:** Diencephalic syndrome, Failure to thrive, Nystagmus, Children, Brain tumors

## Abstract

The aim of our study was to better define the clinical pattern of diencephalic syndrome, a rare but potentially lethal cause of failure to thrive in infancy. Poor weight gain or weight loss, the characteristic presenting feature, often firstly attributed to gastrointestinal or endocrinological or genetic diseases, is secondary to a malfunctioning hypothalamus, caused by a diencephalic tumor. Due to its unexpected clinical onset, diagnostic delay and misdiagnosis are common. We described a case series of 3 children with diencephalic syndrome admitted at our Hospital, over a 5-year period. Furthermore, a narrative review on all pediatric cases published in the last seventy years was performed. Clinical pattern, timing to diagnosis, neuroimaging, management, and outcome were analyzed. Our three cases are singularly described in all clinical and diagnostic findings. Overall, 100 children were selected; all these cases as well as our children presented with failure to thrive: 96% had body mass index or weight-length/height ratio lower than 5^th^ percentile. Vomiting and hyperactivity are reported in 35 and 26% of cases, respectively. The neurological features, mainly nystagmus reported in 43%, may occur late in the disease course. In conclusion, the diagnostic delay is the hallmark of diencephalic syndrome, confirming the lack of knowledge by clinicians. The poor weight gain/loss despite adequate length growth and food intake, especially in children with hyperactivity and good psychomotor development, should alert pediatricians towards this condition, before neurological signs/symptoms occurrence.

## Introduction

Diencephalic syndrome (DS) is a rare but potentially lethal cause of failure to thrive (FTT) in young children, not explained by vomiting and diarrhea, decreased caloric intake or other gastrointestinal causes. It is instead secondary to a malfunctioning hypothalamus, caused by a diencephalic tumor (thalamus/hypothalamus and optic chiasm) [1]. The DS includes clinical characteristics of severe emaciation, and/or crossing down of the 5^th^ percentile of weight, and/or body mass index (BMI) < -2 SD, with normal linear growth and normal intellectual development, in association with central nervous system (CNS) tumors. Since the first description [2], more than a hundred of cases have been published in childhood considering either case series [3–17] and single case reports [18–25]. The clinical features of DS can be categorized in major and minor manifestations. The major features include severe emaciation, despite adequate or slightly decreased caloric intake, locomotor hyperactivity and euphoria, whereas minor features comprise pallor without anemia, hypoglycemia and hypotension [26]. Neurological symptoms, including nystagmus and strabismus, typically have a late appearance delaying diagnostic suspicion; the presence of intermittent vomiting should suggest the diagnosis of obstructive hydrocephalus. Children with DS have no developmental delay; conversely, they may behave in a happy and outgoing manner, which is in contrast to their outward appearance. Such complex non-specific clinical presentation often leads to misdiagnosis. The lack of awareness for this syndrome among pediatricians also contributes to diagnostic delay. Most children, after several pediatric evaluations, undergo an extensive medical work-up with many different specialist consultations. Pediatricians and pediatric specialists should be aware of the possibility of DS when facing children with FTT, especially if height and the caloric intake are normal. Herein, we present three cases with progressive weight loss referred with presumptive diagnosis of malabsorption whose final diagnosis was DS secondary to astrocytoma. Moreover, an extensive narrative review of the literature has been performed, in order to better define the epidemiological and clinical features, and to stress the diagnostic work-up and management of DS.

## Material and methods

**Fig. 1:**
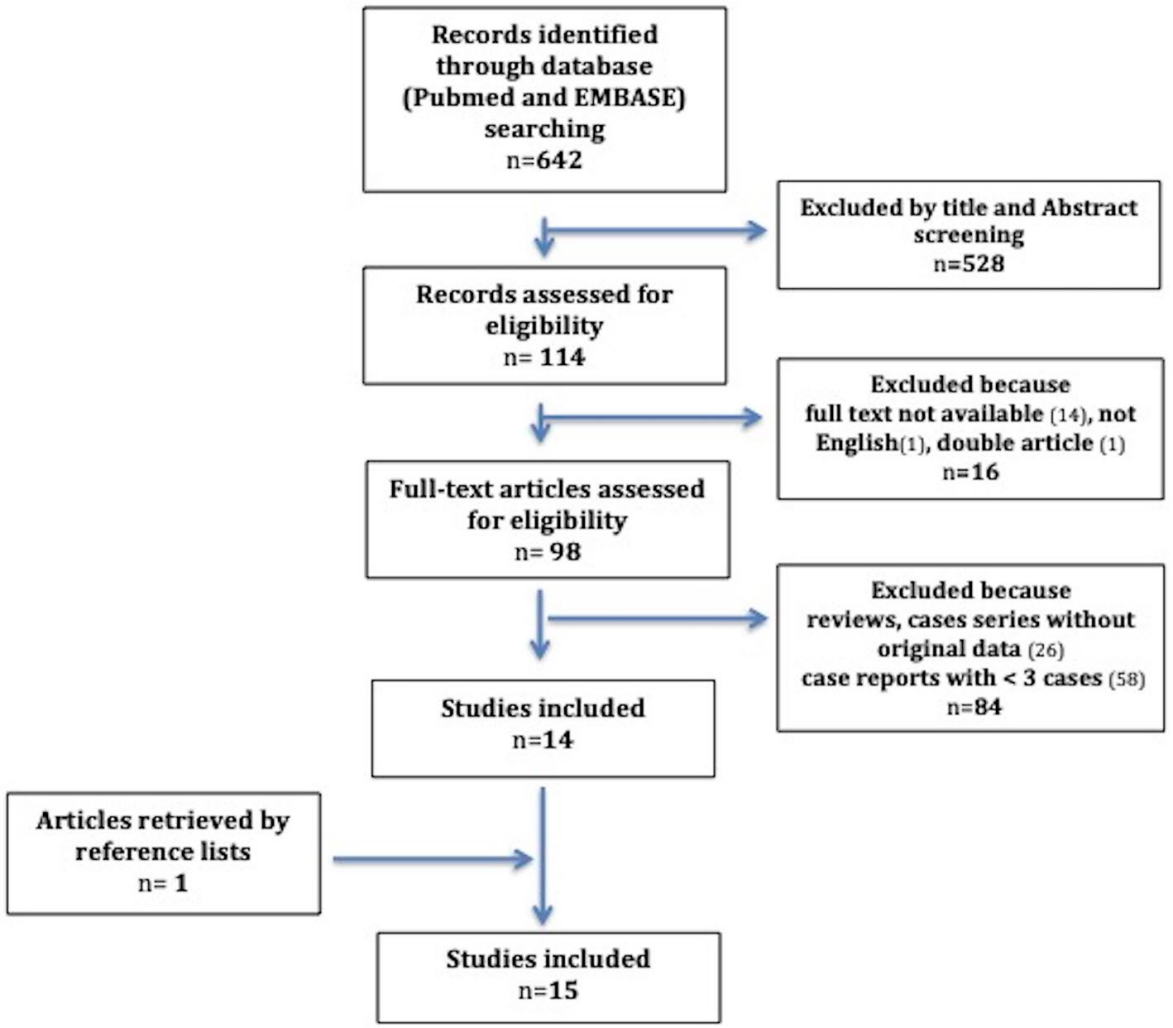
The flow-chart explains the selection method of the articles during the review process

A retrospective chart review of the children admitted to Meyer Children’s Hospital for FTT with further diagnosis of DS over a 5-year period (2015–2019) was performed. Furthermore, an extensive literature review of all reported pediatric cases of DS has been done. Such review was conducted using Embase®, MEDLINE®, MEDLINE®-In Process to identify studies on DS in childhood published as full-text articles from 1951 to December 2020. Databases were searched combining the keywords “Diencephalic Syndrome” or “Russel Syndrome” AND “child” OR “children” OR “infancy”. Articles were included in our study when matching the following eligibility criteria: (1) they provided original data on case series including at least three or more patients, (2) the patients reported were younger than 18 years, (3) they were written in English. Among the 642 records firstly identified through database searching, 528 were excluded by title and abstract screening. Of the remaining 114 articles, 16 were removed because of language (not in English) or the unavailability of the full text. Among the 98 full-text articles assessed for eligibility, 84 were ruled out being case reports, cases series on < 3 cases, or lacking original data. Finally, one article was retrieved by a reference list, reaching 15 articles. A flow-chart detailing the selection method of the articles is shown in the Fig. 1. The three additional cases of DS, identified from our center, have been singularly described.

## Cases description

Epidemiological data, auxological parameters, clinical presentation, magnetic resonance imaging (MRI) findings, management and outcome of three cases admitted to our hospital and reported below are summarized in the Table 1.

**Table 1:** Epidemiological, auxological, clinical data, imaging, pathology, management and outcome of our cases

Sex/age clinical onset (months)	Age at diagnosis (months)	Auxological data			Neurological pattern	Clinical findings	MRI features	Pathology	Chemo-therapy	Surgery	RT	Outcome
			percentile	Z score								
M/18	26	W L W/L HC	3^rd^ 10^th^ -	-1.7 -1.2 -1.4	Irritability Hyperactivity Normal exam	Ematiation Sleeping disorder	suprasellar lesion (44 × 40x38 mm) hypothalamic-pituitary chiasmatic region, hydrocephalus	Low-grade astrocytoma	Carboplatin etoposide	VP derivation	no	alive
F/8	14	W L W/L HC	< 3^rd^ 5^th^ 75^th^	-1.9 -1.6 -1.6	Normal exam Alert	Pale skin emaciation	suprasellar lesion (40 × 36x31 mm) hypothalamic-pituitary region, extended to 3^rd^ ventricle	Low-grade astrocytoma	Carboplatin etoposide vinorelbin	Resection	yes	died
M/8	16	W L W/L HC	10^th^ >> 97^th^ 90^th^	-1.2 4.9 -5	Nistagmus Hyperactivity Normal exam	Pale skin emaciation	Multilobate pseudocystic suprasellar lesion (47 × 38x39 mm) hypothalamic-pituitary and chiasmatic region	Pilomyxoid astrocytoma	Carboplatin Etoposide bevacizumab irinotecan	Debulking	no	alive

*MRI* Magnetic resonance imaging, *M* male, *F* Female, *W* Weight, *L* Length, *W*/*L* Weight/Length, *HC* Head circumference, *VP* Ventriculo-peritoneal, *RT* Radiotherapy

### Case #1

A 26-month-old boy was referred to the Gastroenterology and Nutrition Unit of Meyer Children’s Hospital for persistent failure to gain weight despite adequate caloric intake. He was fine until 18-month-old when he started to wake up scared during the night, walking around the house, and going back to sleep after few hours; subsequently, a deceleration of weight growth, despite an adequate caloric intake, was noticed, in absence of gastrointestinal or other symptoms. Baseline investigations towards malabsorption, including total protein, albumin and vitamins dosages, as well as celiac disease (CD) screening and fecal exams, were negative. Six months later, due to further decrease in weight growth, he was referred to a pediatric endocrinologist: hormonal evaluations including thyroid hormones and abdominal ultrasound were unremarkable. When admitted to our hospital no cardiac, thoracic and abdominal abnormalities were found, as well as the neurological exam and development resulted normal, except for an irritable behavior. In the last month, the child had started to walk around both at home and at the kindergarten also during the day, and he had presented motion sickness during journeys by car. The growth curve showed weight loss (from 25^th^ to < 3^rd^ percentile) started at18 months. His daily dietary intake, assessed through e 3-day-diary administered by our dietitian to the parents, was normal for age. Stool sample tests excluded bacterial, mycotic and parasitic infections; fecal calprotectin and routine blood tests together with nutritional values were normal. In the suspicion of endocranial tumor; abrain MRI was performed; a large supra-sellar lesion (diameters 44 × 40x38 mm) in hypothalamic-pituitary and chiasmatic region, was found, with enhancement after contrast infusion, compressing the cerebral peduncles, Willis’s polygon, chiasmatic optic tracts and third ventricle, with consequential hydrocephalus and lateral ventricles’ dilation. These results led to the final diagnosis of DS, seven months after FTT has begun. Ventriculo-peritoneal derivation (VPD) and mass biopsies were performed. After histological diagnosis of low-grade astrocytoma, the chemotherapy with carboplatin and etoposide was started, with initial weight gain. There was no indication of surgical treatment. The child did not receive either enteral or parenteral nutrition because he well-nourished himself, autonomously; our dietitian enriched his diet with hypercaloric supplements and checked his daily intake. During 6-months follow-up, MRI showed unvaried mass dimensions and impregnation. No endocrinological changes were detected during the follow-up and the child gained weight (25^th^ centile).

### Case #2

A 14-month-old girl was admitted to our hospital for poor weight gain, with normal caloric intake. She was exclusively breast-fed until 6 months, when weaning was started including gluten from the 7^th^ month; no allergies were documented. From the age of 8-month, a slight decrease in growth velocity was highlighted during a scheduled pediatric visit; no symptoms were noticed, except for occasional diarrhea; at evaluation, she presented with good appetite; no episode of vomiting, fever, sweating, or irritability occurred. A first attempt with cow’s milk-free diet resulted unsuccessful. Growth chart evidenced progressive weight loss, falling from 50 to 10^th^ percentile. Baseline investigations, including complete blood count (CBC), hepatic and renal function test, thyroid hormones, electrolytes, total protein and urine analysis were normal. CD screening and stool cultures were negative. At the age of 14 months, due to worsening in FTT, she was hospitalized. On admission, poor general conditions with severe weight loss (< 3^rd^ percentile), length 72 cm (5^th^ percentile) and normal head circumference (75^th^ percentile) were recorded. Pallor and lack of subcutaneous fat were evident. The child was otherwise well, alert, friendly and cooperating, and was achieving appropriate milestone. No abnormalities in neurologic, cardiac, thoracic, lymph nodes and abdominal examination were recorded. Laboratory work-up confirmed normal baseline investigations, excluding infections and intestinal malabsorption. The 3-days-food diary revealed an adequate caloric intake. Stool culture was negative for bacterial, mycotic, and parasitic infections. Peripheral blood smear evidenced normal count blood cell without acanthocytes. Sweat test and autoimmunity markers were negative. Chest X-ray, cardiac evaluation and abdomen echography were normal; hand X-ray confirmed appropriate skeletal age. Ocular examination described pigmented perineural ring without signs of papillary edema or vascular anomalies. During hospitalization, the child did not manifest any eating disorders and stools were normal. No episode of fever, vomiting, excessive sweating, bleeding disorders or neurologic signs appeared. Persisting weight loss along to normal investigations, brain MRI was performed to rule out the possibility of neoplasia, in the hypothesis of DS: a large, enhancing supra-sellar mass (diameters 40 × 36x31 mm) involving hypothalamic-pituitary region, extended to third ventricle with secondary lateral ventricle dilation was found. All these data suggest the final diagnosis of DS. After histologic diagnosis of low-grade astrocytoma, chemotherapy was started. Surgical resection (debulking) and radio therapy (RT) an were also performed. Her weight growth velocity slowly improved, reaching the 10^th^ percentile in 10 months (from < 3^rd^ percentile). Unfortunately, the child died 12 months after diagnosis, due to multiorgan failure and electrolyte imbalance (severe acute hypernatremia) resulting from progressive hypothalamic damage.

### Case #3

A 16-month-old male was hospitalized for severe FTT. He was a full-term new-born from a twin pregnancy complicated by gestational diabetes and systemic hypertension, without signs of intrauterine growth delay. For his first 6 months, he received an adequate caloric intake based on breastfeeding added with formula milk. At the weaning, a transient difficulty in swallowing solid foods was described, quickly normalized with achievement of a various diet. He regularly grew up (weight at 97^th^ percentile) until 8 months; since then, he presented a deceleration in weight gain, falling to 25^th^ percentile, despite a normal height growth, over 8-month-period. The child presented regular diuresis and normal stools; neurodevelopment milestones were properly attained. Blood tests showed mild neutropenia but normal hemoglobin; hepatic and renal function resulted within normal range. The screening for CD was negative as such as the abdomen ultrasound. *Giardia lamblia* was identified at the parasitological exam of stool, and metronidazole was properly administered. Gluten exclusion from diet was attempted for a short period, without any change in growth pattern. At hospital admission, the physical evaluation revealed pale and dry skin, lack of subcutaneous fat, thin and triangular face, sunken anterior fontanelle, and muscle wasting. Auxological parameters measurement revealed weight at 10^th^ percentile, length greater than 97^th^ percentile, head circumference at 90^th^ percentile, and a (BMI) under 3^rd^ percentile. At neurological examination, he showed restlessness, motor hyperactivity, and slight nystagmus. Laboratory work up confirmed mild neutropenia and showed normal hepatic and renal functionality; urine analysis and fecal occult blood test were negative. Dietetic evaluation did not identify any lack in his nutrition with appropriate nutritional daily intake. During the hospitalization, the child appeared hyperactive, kept eating with appetite, without diarrhea or vomiting. A complete hormonal assessment was normal. In the suspicion of lipodystrophy, the detection of specific Single Nucleotide Polymorphism-array, suggested by the geneticist, resulted negative. Suspecting DS, an imaging was performed: MRI documented a large multilobate pseudo-cystic suprasellar lesion (diameters 47 × 38x39 mm) showing positive widespread inhomogeneous enhancement after contrast. The mass involved asymmetrically the hypothalamic-pituitary and chiasmatic region, compressed right side of cerebral peduncles, pons, Willis’s polygon and third ventricle; lateral ventricles dimensions were moderately incremented, without leading to hydrocephalus. These results lead to the diagnosis of DS, 8 months after his weight growth begun decelerating. Histological diagnosis of pilomyxoid astrocytoma was made after biopsy. A low-grade chemotherapy regimen was started with cisplatin and etoposide. This patient is still alive and actively followed up at the time of this report. After 3 years of therapy, the lesion size remains substantially unchanged. He has developed early puberty, which is on hormonal treatment. The weight curve changed from negative to positive after nearly 6 months. One year later, his BMI Z-score was—2.94 (from—5 at the diagnosis) and it continued to slowly increase until the value of 0.01 at the last follow-up, 3 years after the diagnosis.

## Discussion

In 1951, Russel firstly described DS in 12 children with emaciation associated with a loss of subcutaneous fat despite normal intake of calories, nystagmus and hyperkinesia secondary to an intracranial neoplasm involving anterior hypothalamus or optical-chiasmatic glioma [2]. Since then, several studies have been published on this topic. All the case-series describing three or more patients have been reviewed and collected in this article with a final selection of 15 studies, describing complexly 100 children [3–17]. Their epidemiological, auxological and clinical findings are shown in Table 2, where are detailed the data from each single study. The prevalence of the above findings as well as pathology, management and outcome are reported in Table 3.

**Table 2:** Study details of the children with DS from literature review

First author (year)	**Cases** **(% M)**	**Age Onset** **Mean** **(range) *months***	**Age Dx** **Mean** **(range) *months***	**Diagnostic delay** **(range) *months***	**W < 5**^**th**^** c** **available in 71 cases**	**W/L or W/H or BMI < 5**^**th**^** c** **available in 48 cases**	**HC > 97**^**th**^ **available in 25 cases**	**Length or height** **available in 55 cases**		**Clinical presentation** **available in 85 cases**
								< 5^th^ c	> 98^th^ c	
Addy 1972	3 (0)	3 (0–5)	16.3 (9–21)	13.3 (4–20)	2	NR	NR	NR		Nystagmus (1) Vomiting (1) papilledema (2) Visual loss (1) Anorexia (1)
Pelc 1972	3 (100)	3.3 (2–5)	9 (4–17)	5.6 (1–14)	1	NR	2	0	0	Nystagmus (3) Vomiting (3) Visual loss (3) lethargy (1)
Burr 1976	5 (80)	10.8 (4–36)	34.2 (7–120)	23.4 (3–84)	1	NR	1	1	0	Nystagmus (1) Vomiting (5) papilledema (1) Visual loss (2) Neurodevelopmental delay (1) Hyperactivity/happiness (5) Anorexia (1)
DeSousa 1979	12 (50)	8.6 (5–27)	NR	NR	7	NR	NR	NR		Nystagmus (6) Visual loss (10) Hyperactivity (6) Irritability (6)
Namba 1985	3 (66)	10.3 (3–24)	29 (16–45)	18.3 (12–23)	3	NR	NR	NR		Nystagmus (2) Vomiting (1) Visual loss (1) Irritability (1) Hyperactivity/ happiness (1)
Gropman 1998	7 (71)	NR	13.1 (9–20)	NR	5	NR	NR	0	0	NR
Ertem 2000	3 (100)	11.3 (6–22)	16 (6–30)	4.6 (0–8)	3	3	1	0	0	Nystagmus (3) Visual loss (1) strabismus (1) Hyperactivity/happiness (£) Diaphoresis (1)
Fleischman 2005	11 (54)	NR	NR	12.5 (2–33)	10	11	NR	0	1	Nystagmus (3) Vomiting (4) Visual loss (1) Hyperactivity/happiness (2) lethargy (3)
Brauner 2006	11 (45.5)	NR	17.6 (6–108)	NR	NR	9	NR	NR		Nystagmus and strabismus (8) Headache (1)
Densupsoontorn 2011	3 (0)	5.3 (3–7)	12.3 (10–15)	7 (5–9)	3	3	1	1	0	Nystagmus (2) Vomiting (2) Visual loss (1) seizures (1)
Sardi 2012	8 (50)	NR	16.7 (4–60)	NR	6	NR	1	0	0	NR
Hoffmann 2014	11 (36.3)	92 (26–191)	100 (28.8–193.2)	8 (0.5–24)	NR	11	NR	NR		Nystagmus (1) Vomiting (5) Headache (6) polyuria/polydipsia (4) Anorexia (1) seizures (1)
Kilday 2014	9 (55)	NR	16.7 (6.5–32)	NR	6	9	NR	0	1	Nystagmus (6) Vomiting (4) Visual loss (2) strabismus (2) Hyperactivity/happiness (3) Neurodevelopmental delay (3) Anorexia (2)
Kim 2015	8 (12.5)	7 (4–12)	18 (5–38)	11 (1–32)	8	NR	NR	0	0	Nystagmus (1) Vomiting (5) Strabismus (2) Hyperactivity/ happiness (2) Neurodevelopmental delay (2)
Patny 2015	3 (100)	14 (0–36)	75 (66–87)	61.3 (30–87)	2	NR	NR	0	0	Hepatosplenomegaly (1) precocious puberty (1)
TOTAL	100 (50)				57/71	46/48	6/24	2/55	2/55	

*Dx* Diagnosis, *W* weight, *L* Length, *H* Height, *W*/*L*, Weight/Length, *W*/*H* Weight/Height, *BMI* Body mass index, *HC* head circumference, *NR* Not reported, *M* male

**Table 3:** Prevalence of auxological, clinical, pathologic features and therapy in children with DS

**Auxological parameters** **(percentile)**	**%**	Clinical features	**%**	Pathology	**%**	Therapy	**%**	Outcome	%
W < 5^th^	80	Nystagmus	43	Astrocytoma	83	Chemotherapy	49	Survivor	72.5
W/L or W/H or BMI < 5^th^	96	Vomiting	35	Craniopharyngioma	12.5	Radiotherapy	47	died	24.5
HC > 97^th^	25	Hyperactivity/happiness	26			Total resection	17		
L or H < 5^th^	4	Visual loss	26			Partial resection	44		
L or H > 97^th^	4	Strabismus	15						
		Irritability	8						
		Headache	8						
		Neurodevelopment delay	7						
		Anorexia	6						
		Lethargy	5						
		Papilledema	3						
		Seizures	2						

*W* Weight, *L* Length, *H* Height, *W*/*L* Weight/Length, *W*/*H* Weight/Height, *BMI* Body mass index, *HC* head circumference

Considering together the selected reports, among the 100 children reviewed no preponderance in gender has been noted. Surprisingly, some authors had a marked sex prevalence in their series: Kim described seven girls out of eight children (90%) [16], whereas Gropman et al. reported five boys out of seven cases (80%) [8]. The mean age at onset of symptoms was 30.6 months (ranging from 0 to 191): focusing on the cohorts of children with DS due to astrocytoma such as those reported by DeSousa et al., Kim et al., and Patni et al. it was respectively of 8.6, 7, and 14 months [6, 16, 17]. In contrast, when DS is related to craniopharyngioma, as in the cohort of Hoffman et al. [14], the mean age was higher (92 months, range 26–191), and the clinical manifestations often include signs of panhypopituitarism. Actually, in our small miniseries the mean age at symptoms onset was 11.3 months (range 8–18) in agreement with the series of DS and astrocytoma.

All reported patients presented with emaciation, lack of subcutaneous fat up to severe lipodystrophy, muscular hypotrophy, despite an adequate caloric intake. As in our cases, the growth charts document a disproportion between weight loss and length, which is less or no compromised. As regards anthropometric parameters, which have been unevenly available, most cases (57/71, 80%) had a weight at admission lower than 5^th^, while only 9/71 (12.6%) were ≥ 10^th^ percentile. BMI and/or weight/height and or weight/length when reported or calculable, resulted below 5^th^ percentile in 46/48 (96%) children. In contrast, the height, reported in 55 cases, was normal in the majority of patients (51/55, 93%), being only in 2 infants > 97^th^, and < 5^th^ in other 2. As far as it concerns head circumference, it was reported as enlarged > 97^th^ centile, in 6 out of 24 patients in which such parameter was recorded (25%). Clinical presentation was described in all selected articles except the studies of Gropman et al. [8] and of Sardi et al. [13]. Altogether, we obtained clinical information on 85 children. Neurological features of DS are reported, including intracranial hypertension (IH) signs and ocular manifestations. Vomiting and nystagmus are reported in the majority of cases; in particular, nystagmus was found in 37 cases and vomiting in 30 children (43.5 and 35.2%, respectively). Strabismus and visual loss are other ocular findings overall frequently reported: visual loss was referred in 22 (25.8%) cases and strabismus in 13 children (15.2%). Considering altered behavior patterns, hyperactivity and happiness were found in 22 cases (25.8%), irritability in 7 cases (8%), whereas lethargy was reported in 4 cases (5%); sleeping disorders was cited only in one infant. As far as it concerns the attainment of developmental milestones, almost all cases showed a normal psychomotor progress [10], whereas only six patients (7%) showed unspecified neurodevelopmental problems [15, 16]. Pale and dry skin was found in 27 children (27/100, 27%). Other associated rarer signs or symptoms include headache in 7 cases (8%), anorexia in 5 (6%), polyuria and polydipsia in 4 (4.7%), all reported in the cases series of Hoffmann [14]. Other pathological signs as papilledema have been rarely reported (3 cases), precocious puberty and hepatosplenomegaly were noted in two boys described by Patny et al. [17]. All our cases presented with significant weight loss despite appropriate food intake, normal height, whereas neurologic symptoms were limited: patient #1 was collaborative, but sometimes irritable and hyperactive; patient #2 did not show any neurodevelopmental manifestations; in patient #3 hyper-alertness, hyperkinesis and a mild horizontal nystagmus were identified.

The diagnosis of DS is frequently delayed; considering all published reports indicating this finding, the average age at diagnosis was 45.9 months (range from 4 to 193.2 months), with a mean delay from symptoms of 15.2 months (range from 0.5 to 87 months). In our cases, the correct diagnosis was achieved 6 months after initial signs of FTT in the girl #2, and 8 months in cases #1 and #3, with a mean diagnostic delay of 7.3 months, accordingly with Densupsoontorn et al. who found a median interval time of 7 months [12]. Other previous studies showed a slight longer interval time to reach final diagnosis, as in the cohorts of Kim et al. [16] and Addy et al. [3], reporting a median time of 11 and 13 months, respectively. Likewise, in the case series of Brauner et al. [11], the diagnosis was delayed about 12 months on average, and in the cohort of Fleischman about 12.5 months [10]. Sometimes, diagnostic delay might be very high ranging from 18 months in the series of Namba et al. [7] to 6 years in the girl described by Crawford et al. [23].

Hematological abnormalities are very uncommon and non-specific, although the association of some of them along to the clinical aspect should alert towards this condition. In particular, hypoglycemia, hypoproteinemia with hypoalbuminemia have been occasionally found, as well as dyselectrolytemia or increase in urea and creatinine levels due to a possible dehydration [21]. As in Kim’s [16] cohort, in our case series no patient had any significant blood abnormalities suggesting chronic malnutrition. Endocrinological evaluation, usually performed before beginning therapy, includes thyroid hormone levels, IgF-1 concentration, usually normal, growth hormone and cortisol levels, which can be low, normal or even high [10]. Variable hormonal unbalance has been reported, but it was not found in our patients. In the series of Brauner et al., the recurrent reduction in leptin and insulin levels, with concomitant increase in ghrelin and human growth-hormone activity has been reported [11]. Whether these changes are consequent to severe emaciation or responsible for weight loss is still matter of debate. Some authors suggest that metabolism-regulating hormones as leptin [27] and B-lipotropin [28] are directly produced by brain tumors, leading to decrease in subcutaneous fat and excess high release.

To confirm the diagnosis of DS, radiological techniques are required. In the first case series the diagnosis was historically reached through skull X-rays and air-encephalography [3, 6]. Subsequently, computed tomography (CT) scan has been often used to detect cerebral mass [7]. In the last twenty years, MRI has been considered the gold standard and performed as first exam [8, 13, 14]. In our cases MRI was the first radiological investigation performed able to disclose a big suprasellar astrocytoma. In the literature review, histological findings were available in 88 cases: astrocytoma, either pilocytic or pilomyxoid are associated to DS in 83% (73/88) and 20% of these (15/73) were optic gliomas. However, craniopharyngiomas has also been found, although more rarely, accounting for only 12.5% (11/88) of all DS cases.

Focal RT has been associated with excellent disease control but its use is usually avoided or deferred because of the risk of developing neurological impairment. RT, the only treatment associated to mass resection in the children described before twentieth century, was used in 46% of the patients. Since the year 2000 the chemotherapy schedule based on vincristine, carboplatin and/or etoposide, has been successfully introduced. Initially, low-dose chemotherapy regimens have been reported to be effective in hypothalamic low-grade gliomas, with a catch-up growth after 5–8 months from the beginning of the therapy [13]; nowadays, high-dose chemotherapy protocols seem to be the best therapeutic choice. In the review, chemotherapy drugs have been administered in 49% of the cases. The anatomic location of the tumors hinders surgical removal and such approach is not always a feasible option. However, surgery resection has been performed in 61 cases, mostly as partial resection (44 cases), especially in those children with craniopharyngioma [14]. Otherwise, decompressive procedures, as VPD, have been occasionally performed, as in our case #1. Children with DS needed aggressive nutritional support. Parenteral nutrition should be reserved in cases of extreme malnutrition and difficulty in the enteral route. Enteral feeding with a nasogastric tube or parenteral nutrition may provide children unable to feed the nutrients necessary to ensure proper growth, to correct or prevent malnutrition. Nevertheless, oral feeding should be reintroduced as soon as possible, along with a balanced, nutrient-dense oral diet. The dietary intake should be always monitored from the nutritional point of view during the treatment and the subsequent follow-up. Energy and protein were calculated at baseline and periodically thereafter. Despite receiving more than 100% of the nutrient requirement, weight gain varied between patients, possibly related to decrease in tumor size or pressure effects [19].

Most DS cases have been reported in patients with malignant brain tumor associated with high mortality rate. From this review, 76.5% (75/98) of cases are alive; the mortality rate is 24% (23/98), being two cases lost at follow-up. In our series the young girl died after one year from diagnosis, whereas the other two infants at 2-year and 6-month follow-up were alive with weight slightly increased, normal development and tumor stabilization. A variety of clinical sequelae were recorded during the follow-up of the DS patients: the most frequent included significant visual impairment, partial (growth hormone deficiency/precocious puberty as in our case #3) or pan-hypopituitarism, and learning difficulties requiring additional educational support in school (44%), followed by excessive weight gain (33%) [15].

**Fig. 2:**
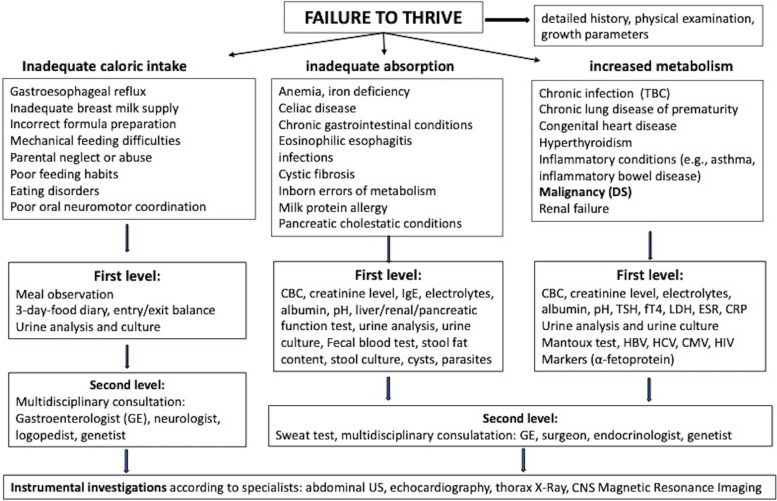
Schematic flow-chart of sequential diagnostic approach to differential diagnosis for children with FTT. CBC: complete blood count, CMV: cytomegalovirus, CNS: central nervous system, CRP: C-reactive protein, DS: diencephalic syndrome ESR: erythrocyte sedimentation rate, FT4: Free thyroxine, GE: gastroenterologist, HBV: hepatitis B virus, HCV: hepatitis C virus, HIV: human immunodeficiency, LDH: lactate dehydrogenase TBC: tuberculosis, TSH: thyroid stimulating hormone, US: ultrasound

DS, also known as Russel Syndrome from the doctor that firstly described it [2], is a rare cause of FTT in young children, often with unfavorable outcome. The exact incidence of DS is unknown; however, although uncommon, it is worthy of attention for pediatricians. This condition represents a possible clinical presentation of suprasellar and third ventricular tumors, developing mostly during infancy or childhood, although it has been rarely described also in adults. Low grade astrocytoma (i.e., pilocytic astrocytoma, and pilomyxoid astrocytoma), is by far the most frequent neoplasm causing DS; however, craniopharyngioma has been also reported in children [14, 26]. Interestingly, DS is sometimes associated to optic pathway gliomas in neurofibromatosis type 1 [29]. Tumors associated to DS are reported to be larger, to occur at a younger age, and to behave more aggressively than similarly located tumors without DS [10]. Underlying pathogenic mechanisms responsible for emaciation have been investigated, but a clear relationship between brain tumors and weight loss has not been completely understood. Cancer development might be responsible for a hyper-catabolic state, leading to increased energy requirement [30]. FTT is the presenting feature in all the cases with DS, along with emaciation and pale skin which are found in almost all children, while height and head are often normal or, even, sometimes increased. Although there is no consensus on the definition of childhood FTT, the term is often used for infants and children with weight < 5^th^ percentile for sex and age. Supporting definitions include weight for length and BMI below the 5^th^ percentile [31]. Being inadequate caloric intake the most common cause of FTT, a detailed nutritional evaluation should be promptly taken. In contrast, an adequate caloric intake is always referred in all patients with DS, especially in the first months of the disease. For pediatricians, the correct diagnostic approach for children with FTT starts with a detailed dietary/growth history, a growth chart creation, and a complete physical examination. This information could help in differentiating cases of inadequate caloric intake, inadequate nutrition absorption, and increased metabolism. Therefore, it is always advisable to begin with the entry/exit calories balance (through a 3-day-food diary and the energy consumption). Subsequently, the pediatrician should request blood tests and consultations with a step-by-step approach: the first level of laboratory exams should include acute phase reactants, CBC, electrolytes, renal and liver function tests, pH, screening for CD, thyroid hormones, urine analysis and culture, fecal blood test, and stool samples for culture, cyst and parasites. Specific testing for cystic fibrosis, food allergies, human immunodeficiency virus infection, tuberculosis or other additional testing should be specific for a suspected diagnosis based on history and physical examination findings. In the second level, a multidisciplinary evaluation is required: gastroenterologist, neurologist, endocrinologist, and genetist may be involved to select other laboratory and/or instrumental tests. A schematic flow-chart of sequential diagnostic approach to differential diagnosis for children with FTT is reported in Fig. 2 [32].

As in our cases, most of children with FTT are seen by pediatric gastroenterologists and their first work-up is usually tailored for the most common pediatric gastrointestinal diseases. The absence of specific clinical signs and laboratory findings allow to rule out the malabsorptive conditions. The physical examination in DS does not usually reveal any signs of underlying chronic or acute illness, out of a state of emaciation. Furthermore, these children may often behave in an alert, happy and outgoing manner. In fact, happiness, hyper-alertness and hyperkinesis are peculiarities of behavior reported in about a quarter of these children. Only rarely children with DS have been referred presenting low appetite up to anorexia and psychological disturbance [33]. The neurological features due to IH are frequently found, being vomiting the most common, followed by headache, instability gait, and, more rarely, papilledema. Ocular manifestations as nystagmus, strabismus, and visual loss, are other common neurological signs related to DS [3–12, 34]. Since both neurological and ophthalmological signs may be late, delay in diagnosis is frequently reported [7, 10, 14–16, 35]. Accordingly, the mean delay time in the published cases is about of 15 months, and in our cases of 11 months. As consequence of diagnostic delay and limited treatment option, final prognosis might be still poor, with a mortality rate of about 25%. Therefore, prompt neuroimaging work-up should be performed in a child with FTT with no evidence of malnutrition and/or malabsorption, before appearing of neurological abnormalities, keeping in mind the possibility of DS. MRI is the gold standard for DS diagnosis but it is often performed when IH signs are already present.

## Conclusions

In the clinical setting of a child with emaciation and FTT, diagnostic delay and misdiagnosis are common. The poor weight gain or loss despite adequate growth in body length and head circumference should alert pediatricians and specialists toward the possibility of DS. Dietary anamnesis commonly highlights adequate caloric intake, and this should be considered an element of high suspicion, especially in children with euphoric mood. Our intent is to increase the awareness of DS not only in pediatricians but also in pediatric gastroenterologists, and endocrinologists who might have to deal with children presenting with severe weight loss despite a normal height, a good psychomotor development, an adequate food intake and a normal absorptive small intestine function.

When an adequate caloric intake is referred, in a child with normal length and normal neurological development but with irritability and hyperactivity, even without neurological signs/symptoms, consider the possibility of neurological imaging in order to exclude DS.

## Data Availability

The datasets used and/or analyzed during the current study are available from the corresponding author on reasonable request.

## Abbreviations

DS: Diencephalic syndrome
FTT: Failure to thrive
CNS: Central nervous system
MRI: Magnetic resonance imaging
CBC: Complete blood count
CD: Celiac disease
VPD: Ventriculo-peritoneal derivation
BMI: Body mass index
IH: Intracranial hypertension
CT: Computed tomography
RT: Radiotherapy

## References

[1] Murphy AM, Drumm B, Brenner C, Lynch SA (2006). Diencephalic cachexia of infancy: Russell’s syndrome. Clin Dysmorphology.

[2] Russell A (1951). A diencephalic syndrome of emaciation in infancy and childhood. Arch Dis Child.

[3] Addy DP, Hudson FP (1972). Diencephalic syndrome of infantile emaciation: analysis of literature and report of further 3 cases. Arc Dis Child.

[4] Pelc S, Flament-Durand J (1973). Histological evidence of optic chiasma glioma in the "diencephalic syndrome". Arch Neurol.

[5] Burr IM, Slomin AE, Danish RK, Gadoth N, Butler IJ (1976). Diencephalic syndrome revisited. J Pediatr.

[6] DeSousa AL, Kalsbeck JE, Mealey J, Fitzgerald J (1979). Diencephalic syndrome and its relationship to optico-chiasmatic glioma: review of twelve cases. Neurosurgery.

[7] Namba S, Nishimoto A, Yagyu Y (1985). Diencephalic syndrome of emaciation (Russell's syndrome). Long-term survival Surg Neurol.

[8] Gropman AL, Packer RJ, Nicholson HS, Vezina G, Jakacki R, Geyer R (1998). Treatment of diencephalic syndrome with chemotherapy. Cancer.

[9] Ertem D, Acar Y, Alper G, Kotiloglu E, Pehlivanoglu E (2000). An uncommon and often overlooked cause of failure to thrive: diencephalic syndrome. JPGN.

[10] Fleischman A, Brue C, Poussaint TY, Kieran M, Pomeroy SL, Goumnerova L (2005). Diencephalic syndrome: a cause of failure to thrive and a model of partial growth hormone resistance. Pediatrics.

[11] Brauner R, Trivin C, Zerah M, Souberbielle JC, Doz F, Kalifa C (2006). Diencephalic syndrome due to hypothalamic tumor: a model of the relationship between weight and puberty onset. J Clin Endocrinol Metab.

[12] Densupsoontorn N, Jirapinyo P, Likasitwattanakul S, Sanmaneechai O, Sanpakit K, Surachatkumtonekul T (2011). Diencephalic syndrome due to astrocytoma in three infants with failure to thrive. Pediatr Int.

[13] Sardi I, Bresci C, Schiavello E, Biassoni V, Fratoni V, Cardellicchio S (2012). Successful treatment with a low-dose cisplatin–etoposide regimen for patients with diencephalic syndrome. J Neurooncol.

[14] Hoffmann A, Gebhardt U, Sterkenburg AS, Warmuth-Metz M, Müller HL (2014). Diencephalic syndrome in childhood craniopharyngioma–results of German multicenter studies on 485 long-term survivors of childhood craniopharyngioma. J Clin Endocrinol Metab.

[15] Kilday JP, Bartels U, Huang A, Barron M, Shago M, Mistry M (2014). Favourable survival and metabolic outcome for children n with Diencephalic syndrome. J Neurooncol.

[16] Kim A, Moon JS, Yang HR, Chang JY, Ko JS, Seo JK (2015). Diencephalic syndrome: a frequently neglected cause of failure to thrive in infants. Korean J Pediatr.

[17] Patni N, Alves C, von Schnurbein J, Wabitsch M, Tannin G, Rakheja D (2015). A novel syndrome of generalized lypodystrophia associated with pilocytic astrocytoma. J Clin Endocrinol Metab.

[18] Greenes D, Woods M (1996). Case report: a 4-month-old boy with severe emaciation, normal linear growth, and a happy affect. Curr Opin Pediatr.

[19] Stival A, Lucchesi M, Farina S, Buccoliero AM, Castiglione F, Genitori L (2015). An infant with hyper-alertness, hyperkinesis, and failure to thrive: a rare diencephalic syndrome due to hypothalamic anaplastic astrocytoma. BMC Cancer.

[20] Tosur M, Tomsa A, Paul DL (2017). Diencephalic syndrome: a rare cause of failure to thrive. BMJ Case Rep.

[21] Roncall BR, Bellapukonda S, Mohanty CR (2019). Diencephalic syndrome: an anaesthetic challenge. BMJ Case Rep.

[22] Conway M, Ejaz R, Kouzmitcheva E, Savlov D, Rutka JT, Moharir M (2016). Child neurology: diencephalic syndrome-like presentation of a medullary brainstem tumor. Neurology.

[23] Crawford JR, Shayan K, Levy M (2013). Delayed presentation of diencephalic syndrome associated with leptomeningeal dissemination in a child. BMJ Case Rep.

[24] Curran MA, Madavhan VL, Caruso PA, Ebb DH, Williams EA (2017). Case 31–2017. A 19-month-old girl with failure to thrive. N Engl J Med.

[25] Wagner LM, Myseros JS, Lukins DE, Willen CM, Packer RJ (2018). Targeted therapy for infants with diencephalic syndrome: a case report and review of management strategies. Pediatr Blood Cancer.

[26] Satyarthee GD, Chipde H (2018). Diencephalic syndrome as presentation of giant childhood craniopharingioma: a management review. J Pediatr Neurosci.

[27] Velasco P, Clemente M, Lorite R, Ventura MC, Gros L, Sanchez de Toledo  J (2014). The role of leptin in diencephalic syndrome. Pediatrics.

[28] Drop SL, Guyda HJ, Colle E (1980). Inappropriate growth hormone release in the diencephalic syndrome of childhood: case report and a 4-year endocrinological follow-up. Clin Endocrinol (Oxf).

[29] Santoro C, Perrotta S, Picariello S, Scilipoti M, Cirillo M, Quaglietta L (2020). Pre-treatment endocrine disorders due to optic pathway gliomas in paediatric neurofibromatosis type 1: multicentre study. J Clin Endocrinol Metab.

[30] Vlachopapadopoulou E, Tracey KJ, Capella M, Gilker C, Matthews DE (1993). Increased energy expenditure in a patient with diencephalic syndrome. J Pediatr.

[31] Shields B, Wacogne I, Wright CM (2012). Weight faltering and failure to thrive in infancy and early childhood. BMJ.

[32] Homan GJ (2016). Failure to thrive: a clinical guide. Am Fam Physician.

[33] Distelmaier F, Janssen G, Mayatapek E, Schaper J, Göbel U, Rosenbaum T (2006). Disseminated pilocytic astrocytoma involving brain stem and diencephalon: a history of atypical eating disorder and diagnostic delay. J Neurooncol.

[34] Dhannawat SS, Nageswara-Rao AA, Brodsky MC (2015). Nystagmus in an emaciated infant. JAMA Ophthalmol.

[35] Huber J, Sovinz P, Lackner H, Mokry M, Eder H, Urban C (2007). Diencephalic syndrome: a frequently delayed diagnosis in failure to thrive. Klin Pediatr.

